# Supplementary material for: Habitat suitability mapping and landscape connectivity analysis to predict African swine fever spread in wild boar populations: A focus on Northern Italy
Source: PLoS One. 2025 Jan 30;20(1):e0317577. doi: 10.1371/journal.pone.0317577 (PMC11781678; doi:10.1371/journal.pone.0317577)
Supplement: S1 Table — Variables taking into account distance from environmental features are shortened with "Dist. from”, while those considering topological patterns with "Top.". Variable references are available in S2 Table. (PDF) [file pone.0317577.s001.pdf]

S1 Table. Environmental variables included in the species distribution models (SDM) for each scenario, after testing for collinearity and multicollinearity. Variables taking into account distance from environmental features are shortened with "Dist. from”, while those considering topological patterns with "Top.". Variable references are available in S2 Table.

Variables	Overall	Winter	Spring	Summer	Autumn
Altitude	x	x	x	x	x
Slope	x	x	x		x
Precipitation		x	x		x
Temperature					
NDVI[a]					
Bare Coverage	x	x	x	x	x
Herbaceous Coverage					
Tree Coverage	x			x	
Population Density	x	x	x	x	x
Dist. from Crop Areas				x	
Dist. from Forest	x	x	x	x	x
Dist. from Highways	x	x	x	x	x
Dist. from Lakes	x	x	x	x	x
Dist. from Rivers	x	x	x	x	x
Dist. from Parks	x	x	x	x	x
Dist. from Urban Areas					
Road Density	x	x	x	x	x
Top. Aspect	x	x	x	x	x
Top. Diversity					
Top. Position	x	x	x	x	x
**Total variables**	**13**	**13**	**13**	**13**	**13**

[a] Normalized Difference Vegetation Index
