# Supplementary material for: Habitat suitability mapping and landscape connectivity analysis to predict African swine fever spread in wild boar populations: A focus on Northern Italy
Source: PLoS One. 2025 Jan 30;20(1):e0317577. doi: 10.1371/journal.pone.0317577 (PMC11781678; doi:10.1371/journal.pone.0317577)

Appendix 1

For each scenario (overall, winter, spring, summer, autumn), response curves of the computed Gradient Boosted Machine ensemble model are shown. GBM ensemble model was computed through the median of all models with ROC greater than 0.8, merging all runs. The red line describes the trend in the probability of wild boar presence as the predictor variable increases, while the grey envelope shows the confidence interval.

Overall Scenario

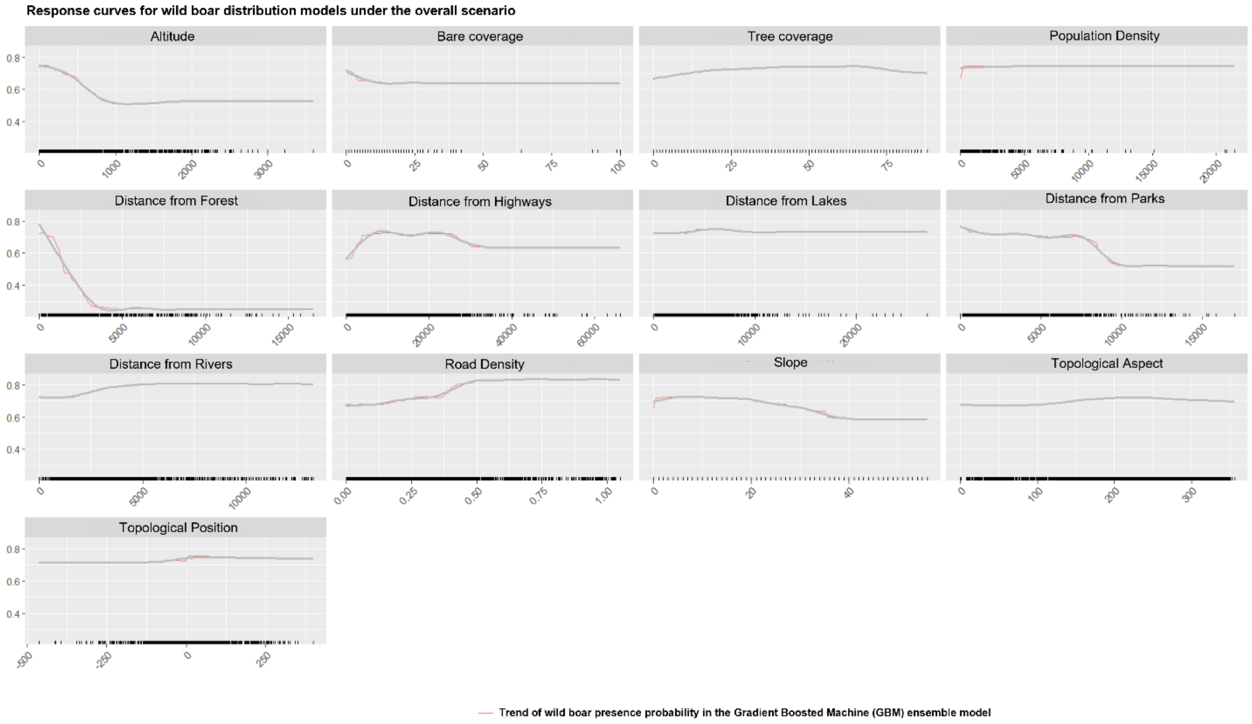

Winter Scenario

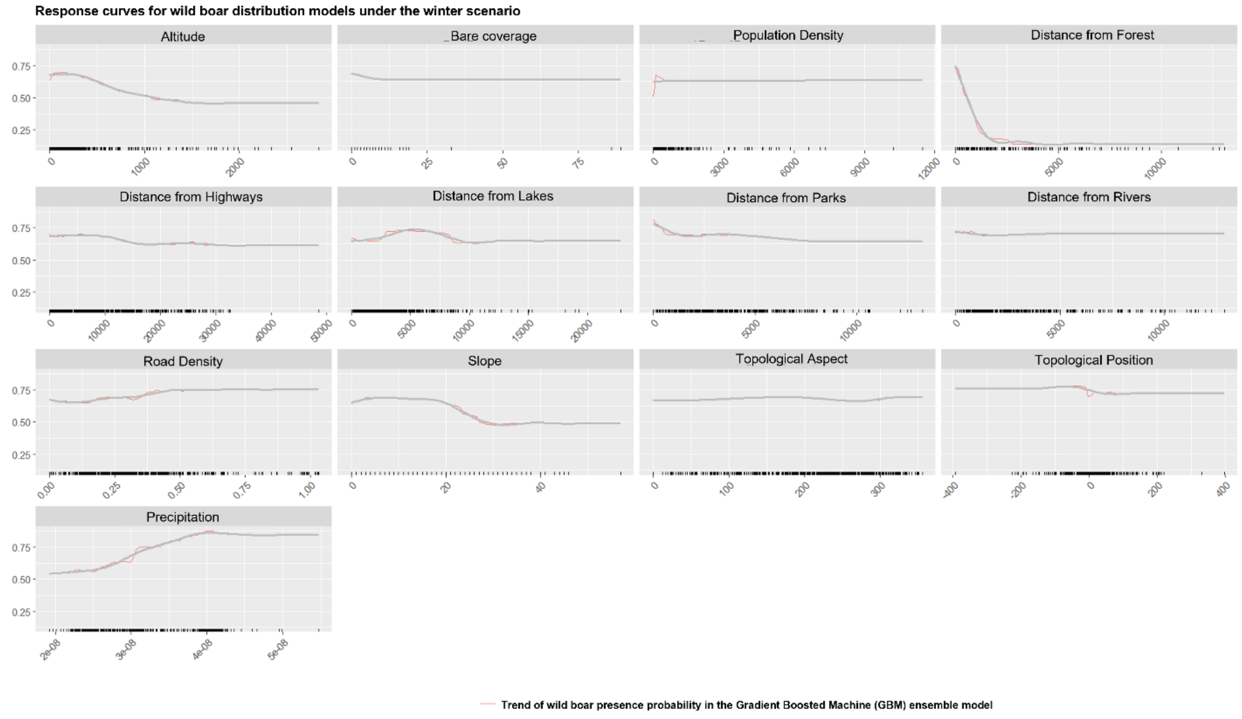

Spring scenario

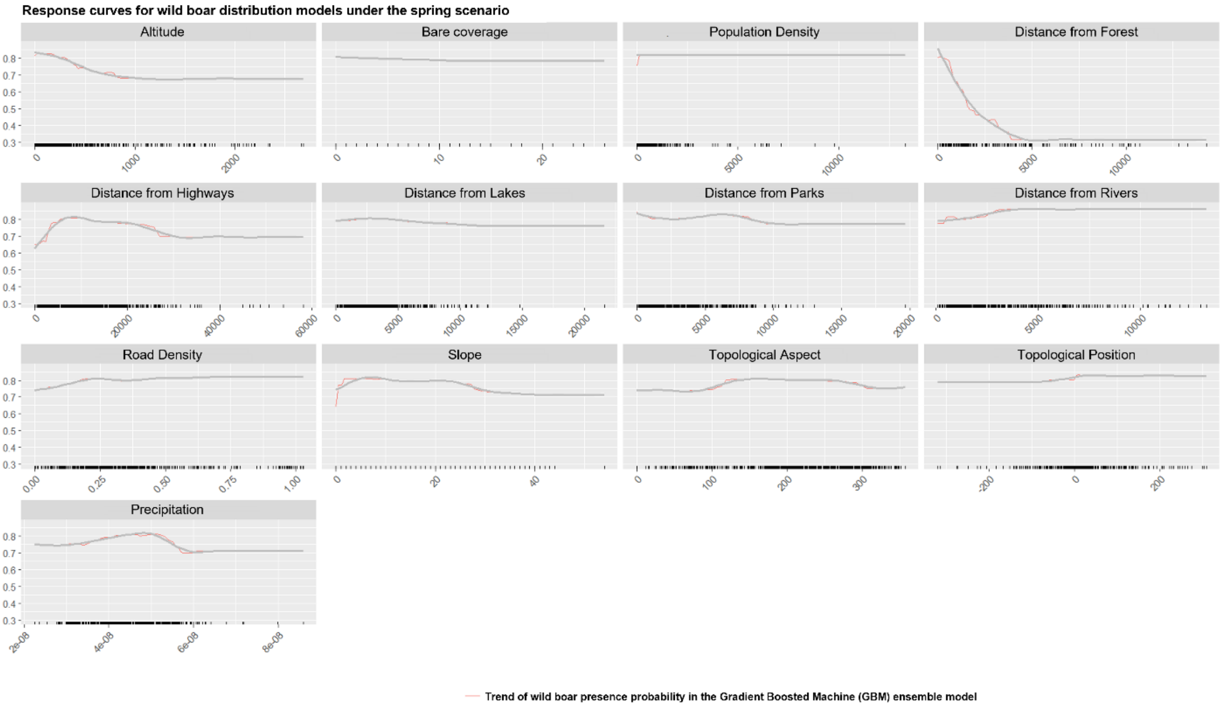

Summer scenario

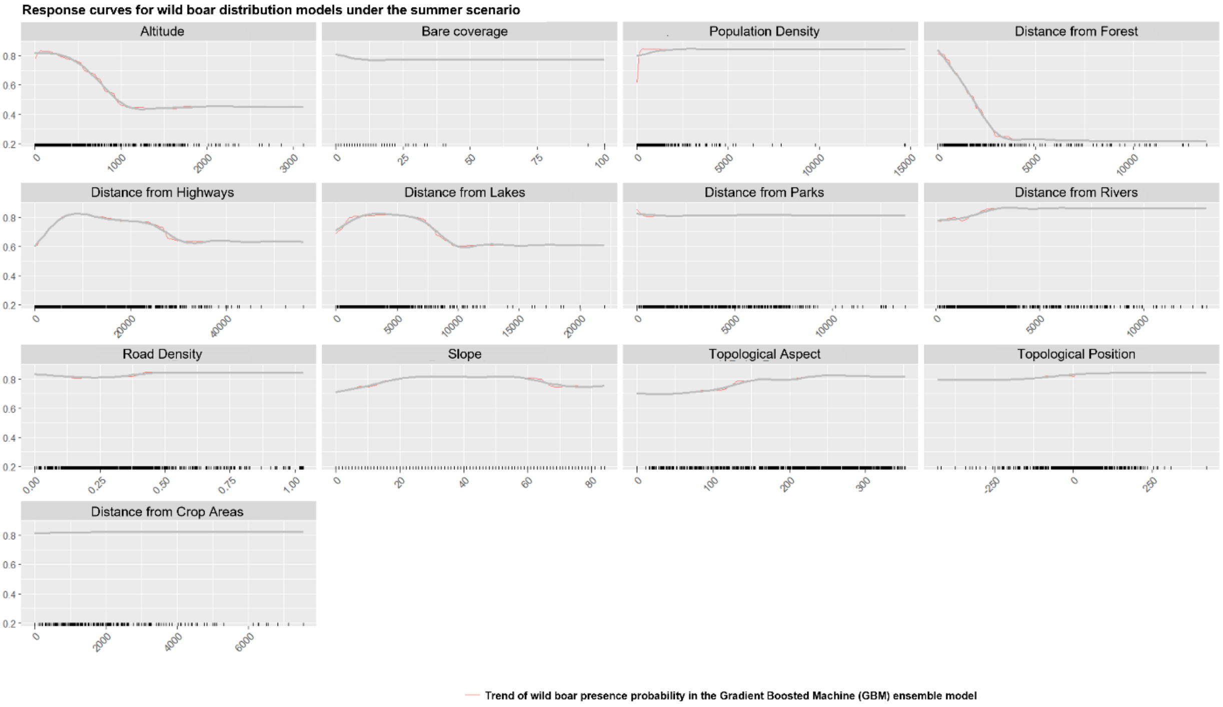

Autumn scenario

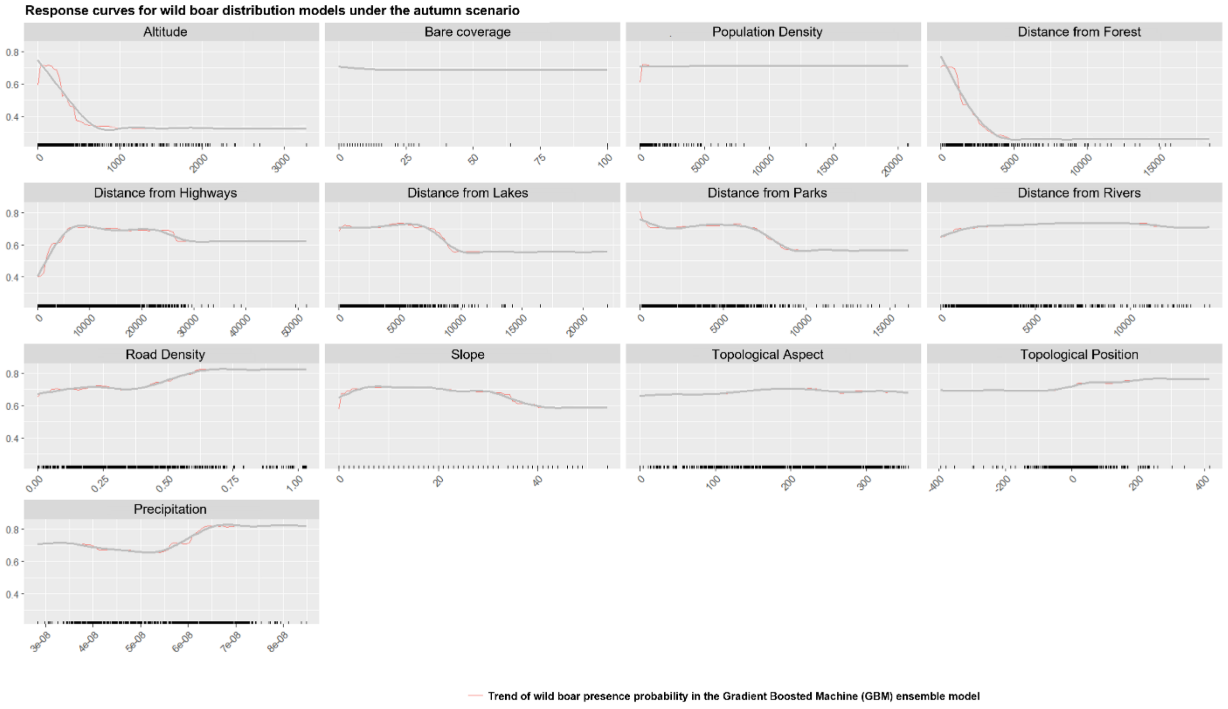

Supplement: S1 Appendix — (PDF) [file pone.0317577.s004.pdf]
