# Supplementary figures and images for: Habitat suitability mapping and landscape connectivity analysis to predict African swine fever spread in wild boar populations: A focus on Northern Italy
Source: PLoS One. 2025 Jan 30;20(1):e0317577. doi: 10.1371/journal.pone.0317577 (PMC11781678; doi:10.1371/journal.pone.0317577)

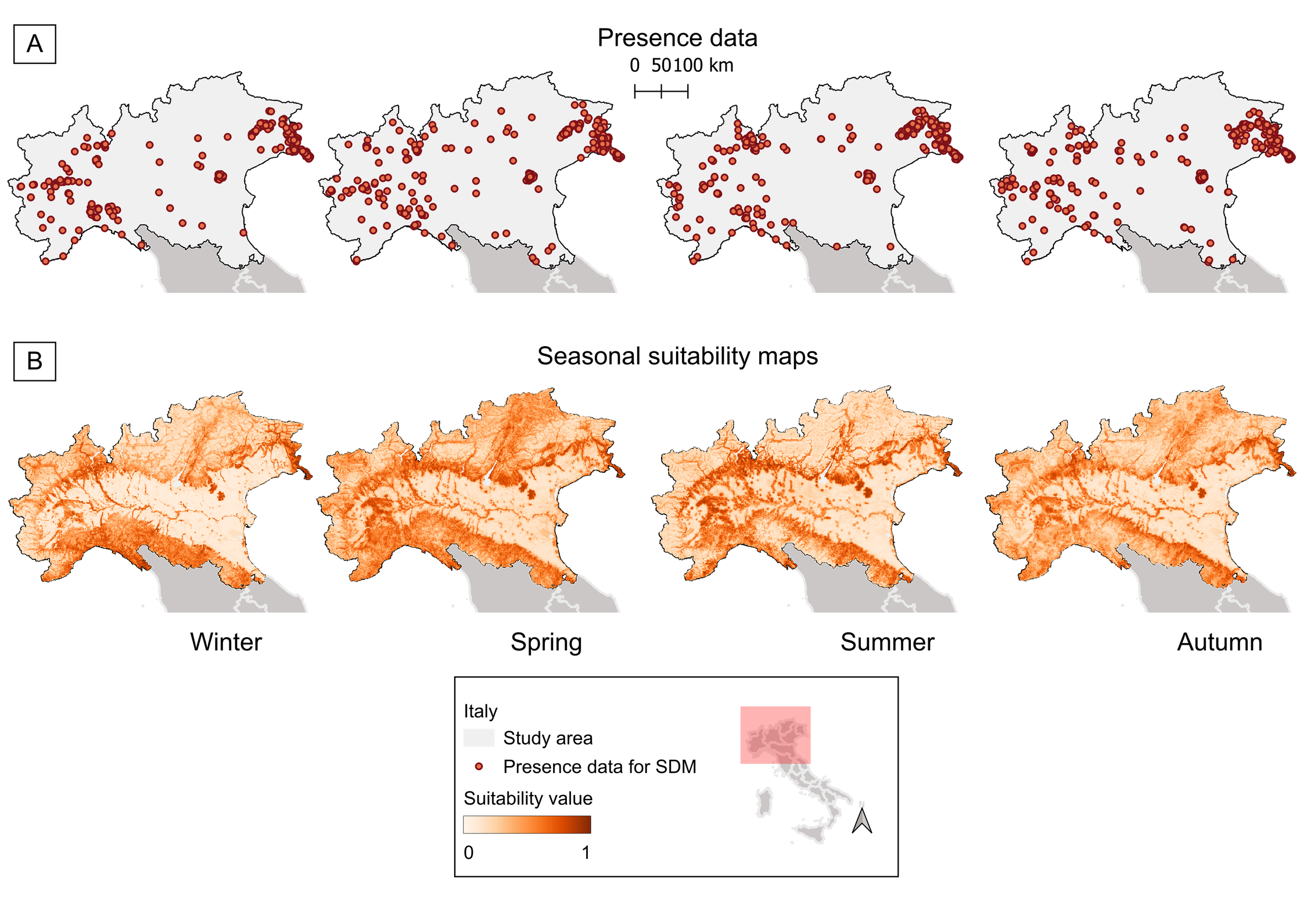

Supplement: S1 Fig — (A) Distribution of occurrence records included in the final model (red points) by season. (B) Maps describing habitat suitability for the presence of wild boars in Northern Italy referring to environmental conditions by season. Base map of Italy based on the 2024 regional administrative boundaries from the Italian National Institute of Statistics (https://www.istat.it/). (TIF) [file pone.0317577.s005.tif]

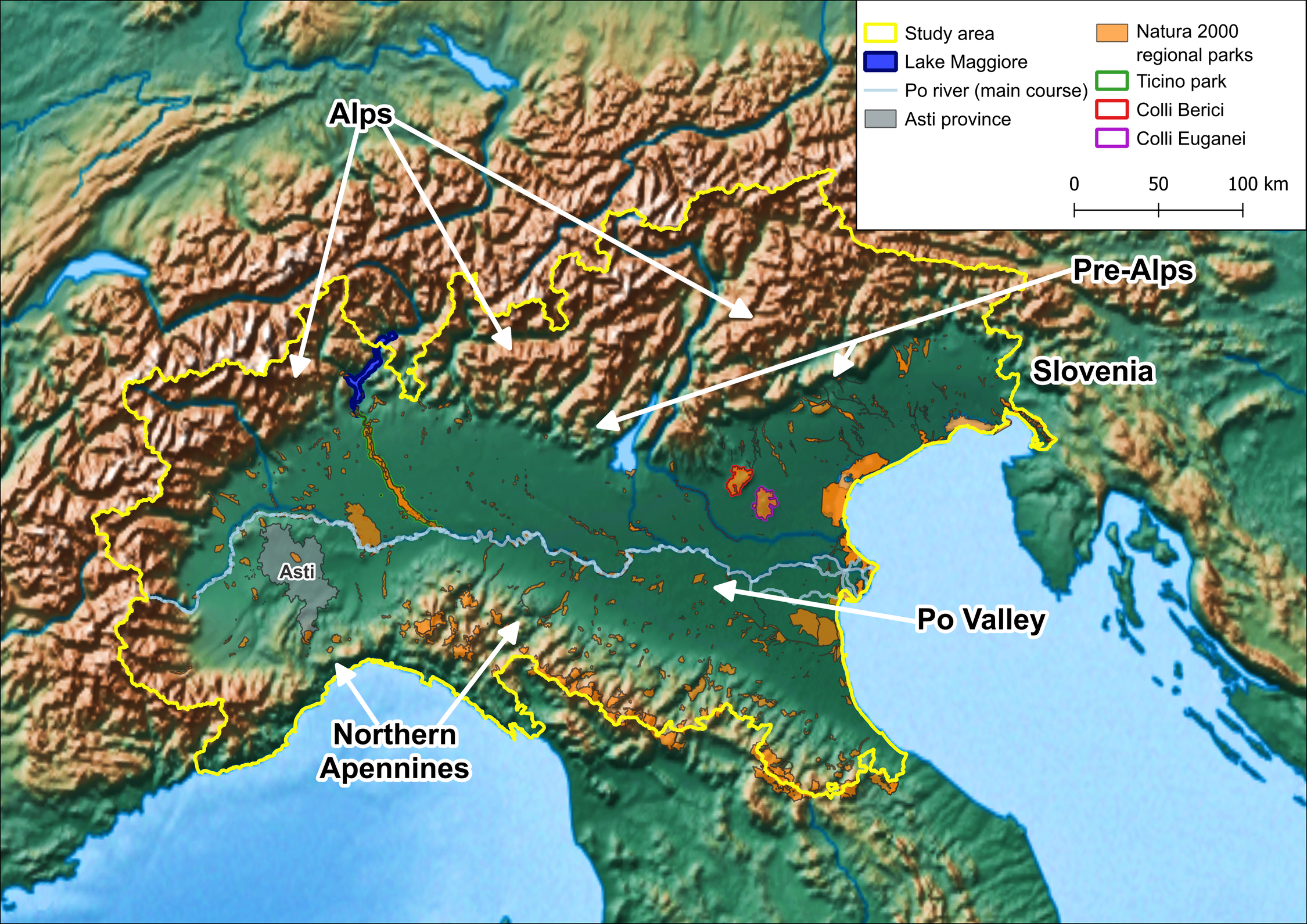

Supplement: S2 Fig — Base map made with Natural Earth. Study area and Asti province based on the 2024 administrative boundaries from the Italian National Institute of Statistics (https://www.istat.it/). Lake Maggiore, Po River, regional parks details made from vector files available on the National geoportal of the Italian Ministry of Environment and Energy Security (https://gn.mase.gov.it/) for illustrative purpose only. (TIF) [file pone.0317577.s006.tif]
