# Supplementary material for: Habitat suitability mapping and landscape connectivity analysis to predict African swine fever spread in wild boar populations: A focus on Northern Italy
Source: PLoS One. 2025 Jan 30;20(1):e0317577. doi: 10.1371/journal.pone.0317577 (PMC11781678; doi:10.1371/journal.pone.0317577)
Supplement: S1 File — (PDF) [file pone.0317577.s007.pdf]

S1 Files. Main code scripts used in the workflow are available at https://doi.org/10.5281/zenodo.14639171. Link to Gitlab repository:

https://gitlab.com/giuliafaustini1/habitat-suitability-mapping-and-landscape-connectivity-analysis-to-predict-african-swine-fever-spread-in-wild-boar-population
