# Supplementary material for: Habitat suitability mapping and landscape connectivity analysis to predict African swine fever spread in wild boar populations: A focus on Northern Italy
Source: PLoS One. 2025 Jan 30;20(1):e0317577. doi: 10.1371/journal.pone.0317577 (PMC11781678; doi:10.1371/journal.pone.0317577)
Supplement: S2 File — (PDF) [file pone.0317577.s008.pdf]

S2 Files. Suitability maps, connectivity map and binary risk maps are available at https://doi.org/10.5281/zenodo.14637229.
