# Supplementary material for: Habitat suitability mapping and landscape connectivity analysis to predict African swine fever spread in wild boar populations: A focus on Northern Italy
Source: PLoS One. 2025 Jan 30;20(1):e0317577. doi: 10.1371/journal.pone.0317577 (PMC11781678; doi:10.1371/journal.pone.0317577)
Supplement: Attachment — Submitted filename: Response to Reviewers.docx [file pone.0317577.s009.docx]

**Response to Academic Editor**

Dear Authors,

the topic of the manuscript is interesting, and you are working with a valuable dataset. However, as highlighted by the reviewers, all sections require improvements. While ensemble modeling can be a powerful tool, it may also lead to misinterpretations if not applied properly. Simply adding more statistics does not necessarily result in better outcomes. Morevoer, since the main objective of your manuscript focuses on connectivity and the role of corridors, these aspects should be discussed in greater detail. I encourage you to carefully address the reviewers' constructive comments.

A: We thank the academic editor for the positive feedback and for highlighting key areas for improvement. We have addressed each of the reviewers' comments and incorporated their suggestions into the revised manuscript. Specifically, we have better clarified our approach, clarifying its application and limitations to avoid any potential misinterpretations. Additionally, as recommended, we have enriched the discussion of connectivity and corridor roles, detailing how these features impact wild boar movement and potential disease spread across the landscape.

Moreover, the following adjustments were made in figures, tables based on the reviewers' valuable suggestions:

- **Figure 3**: We now present only the overall habitat suitability map, with seasonal results provided separately as **S1 Fig**.
- **Figure 4**: As suggested by Reviewer 1, we included a zoomed-in view of infected areas in the connectivity map to enhance focus on regions most affected by African swine fever.
- **S2 Fig**: We added a new figure illustrating detailed geographical context, addressing Reviewer 2’s request.
- **Table 1**: We clarified the methodology by adding a description of the standardization process for the raster data, as suggested by Reviewer 1.
- **Data Availability**: Following the suggestions for data transparency, we have made all associated scripts (S1 Files) and original raster files (S2 Files) available via GitLab and Google Drive links, with the files set to be publicly accessible upon acceptance of the manuscript.
- **Corresponding Author Contact**: We included an additional email for the corresponding author to accommodate a potential upcoming transition to a new institutional affiliation at the University of Padova.
- **Funding Statement**: the funding acknowledgment now reads: "The research project was made possible by the financial support provided to G.F. by the Società Italiana di Patologia ed Allevamento dei Suini (SIPAS, <https://www.sipas.org/chi-siamo/>) and by the Erasmus + for traineeship mobility program (<https://erasmus-plus.ec.europa.eu/opportunities/opportunities-for-individuals/students/traineeships-abroad-for-students>). The funders had no role in study design, data collection and analysis, decision to publish, or preparation of the manuscript. There was no additional external funding received for this study."

We hope to have addressed each of the reviewers' feedback comprehensively, and we thank them for helping us in refining the manuscript. We look forward to your further feedback.

**Response to Reviewers**

**Reviewer #1:** I appreciate the effort of the authors to pull together this information, however, evaluating all possible models in the Biomod2 package does not seem like an ideal study design. Even more confusing is the reason for choosing GBM as more preferred. The authors have a suitable dataset to use a few models for their purposes. With their knowledge and data available, a model or even a few could be chosen but certainly 11 models are not needed to be evaluated. Then we have to delve into the evaluation criteria for why the other 8 models are not suitable which just detracts from the focus of your study design..predicting ASF across the landscape.
A: We thank the reviewer for the valuable comments, and hope that our revisions will address his/her concerns effectively. Please see below.

In my comments below, I tried to assist the authors on how to address some of these issue so their objectives and methods are more clear and support their findings with some specific comments by line:
A: We would like to sincerely thank the reviewer for the insightful and constructive comments. We truly appreciate the time and effort taken to provide suggestions on our manuscript.

Title: Habitat suitability mapping and landscape connectivity analysis to predict African swine fever spread in wild boar population: a focus on northern Italy. I believe populations should be plural or place an “a” before “wild boar” if authors are suggesting this is one single wild population throughout their study site where records are documented?

A: We thank the reviewer for the suggestion. We modified the title accordingly, to improve it clarity.

Introduction

Lines 44 and 51: Defining African swine fever as ASF and African swine fever virus as ASFV seems confusing and unnecessary. The authors then go into ASFV genotype I and ASFV genotype II but reference ASF to discuss the disease in general terms. It is difficult for the reader to know which is meant or which is most appropriate each time either are used. I would suggest do not define ASFV and refer to these as genotype I and genotype II each time it is needed. Then use ASF when discussing the disease in general. Furthermore, there is no mention of these in the remainder of the manuscript aside from ASF.

A: We thank the reviewer for the valuable suggestion to improve the clarity of our manuscript. Following reviewer’s suggestion, the introduction was structured presenting at the beginning only the disease as caused by the virus, and only later the virus with its genotypes. We preferred to keep the distinction between genotype I and II, to present the different epidemiological scenarios of Sardinia Island compared to the rest of Europe and Italy (mainland). However, we acknowledge reviewer observation that ASFV genotypes I and II are not referenced in the remainder of the manuscript. In the revised version, we ensured that any reference to ASF cases specifies the genotype II, including a general statement in the “model evaluation” section in line 333-334.

Lines Line 116: “different wild boar presence data types” is very confusing as written. I would suggest “Presence/absence data for wild boar differs considerably, although often in relation to a common/similar set of environmental variables describing topography, climate, human disturbance and land cover [29].” The authors then need to cite more than one manuscript if these types do differ in the literature.
A: We thank the reviewer for the suggestion. We modified the manuscript accordingly, citing also more articles.

Line 122: ENET needs to be spelled out prior to using an acronym because most won’t be familiar with this term.
A: We acknowledge that this term may appear to be an acronym, but it is, in fact, the official name of the project/consortium. Unfortunately, there is no acronym definition provided by the European Food Safety Authority (EFSA). Instead, EFSA defines *ENETWILD* as an international network of wildlife professionals, which we had described in line 142-143 as "an international network of wildlife professionals supported by the European Food Safety Agency." We apologize for any confusion and hope this clarification resolves the issue.

Line 128: “..dispose of..” I am not sure what this means? This entire sentence is very confusing and might be better split into 2 separate sentences.

A: We apologize for the misleading sentence. The sentence has been revised to enhance its clarity. Specifically, we have replaced "dispose of" with "would benefit from" and have split the sentence into two parts for better readability (see lines 148-151).

Materials and methods

Line 177: Supplement Table S1. This information is often overlooked and I appreciate this table. This is the most complete and detailed variable table I have ever reviewed, thank you for this great summary table!

A: We sincerely thank the reviewer for appreciating our work and effort! We wanted to provide something helpful for improving the reproducibility of our methods.

Lines 170-188: A few points of clarity would be helpful in this section.

1. Density – are these within a 100 x 100 m raster that lines up with all of your rasters? If not, how do you get percentage assigned to each raster cell for bare, herbaceous, and tree cover?

A: Yes, all the rasters were standardized to 100x100m grid cells using a bilinear sampling method , including the coverage layers, as explained in lines 200-203. To avoid confusion, we add this specification also in the description of each variable in S1 Table.

2. Bilinear sampling of 30x30 m raster to derive 100x100m rasters? If so, this should be presented in your “percentage of forest” in your S1 Table.
A: All rasters were standardized to 100x100m grid cells, as explained in lines 200-203 in material and methods section. We are not sure what the reviewer refers to as there aren’t any variables that were standardized from 30x30 m raster to derive 100x100m raster. However, to avoid confusion we add the specification “Bilinear sampling method was applied to standardize the variable as raster with a pixel size of 100 m x 100 m, over an extent equal to the study area.” also in the description of each variable in S1 Table. We thank the reviewer for the opportunity to clarify better our methodology to the reader.

3. By “overall mean” do you mean “annual mean” for each year from 2014-2023 or a single static 10 year mean from all years: 2014-2023?
a. A mean for a 10 year window, if that is what was done, is of no value so please be clear here. Each year of wild boar data should be matched to variables when available. Specifically, NDVI could have massive fluctuations from year to year so a 10 year average does not seem acceptable in any way?

A: We thank the reviewer for highlighting this important point. To clarify, when we refer to "overall mean," we are specifically addressing environmental/climatic variables, which represent long-term environmental patterns rather than short-term weather fluctuations. In niche modeling, the objective is to capture stable climatic conditions that shape habitat suitability over extended periods, rather than ephemeral weather-driven variations. Generally, climatic data spanning even 30 years is standard practice for such analyses.

Therefore, we respectfully disagree with the suggestion that a 10-year mean holds no value in this context. We believe that our approach is well-suited to the scale and aims of our study, focused more on the general suitability of the landscape for wild boars (which is a highly adaptable species), rather than the year-to-year variability that might arise from short-term shifts.
Our approach, which involves calculating an overall mean for the 2014–2023 period, was chosen deliberately to smooth out short-term fluctuations and provide a more stable representation of the environmental/climatic conditions affecting wild boar populations over time. While annual data may indeed capture year-to-year variability, such as NDVI fluctuations, our primary focus was on long-term trends and consistent drivers of wild boar presence. Using a 10-year mean allowed us to generalize the broader environmental context, providing a more robust measure of habitat conditions that is less sensitive to short-term fluctuations. Furthermore, it is not uncommon in ecological and environmental studies to use multi-year averages for environmental/climatic variables, particularly considering data availability and when the goal is to account for general patterns over an extended time frame rather than year-to-year variability (here some examples: <https://doi.org/10.1371/journal.pone.0193295>, <https://doi.org/10.1371/journal.pone.0176339>, <https://doi.org/10.1007/s10980-021-01371-y>).

That said, we acknowledge the limitations of our method, particularly as pointed out by the reviewer and that using year-specific NDVI could enhance temporal precision in future studies focused on annual fluctuations and dynamics. We added this points in the manuscript in line 208-210 and 514-522.

4. What are the “seasons” based on? Some citations for seasons in northern Italy would be advisable.
A: The seasons were defined based on the meteorological convention, which divides the year into four three-month periods: December, January, and February for winter; March, April, and May for spring; June, July, and August for summer; and September, October, and November for autumn. This method does not account for shorter units of time, such as weeks or days, and was used for its simplicity and convenience. Unlike the astronomical definition, which aligns the seasons with solstices and equinoxes, the meteorological definition directly reflects temperature and weather patterns, which are more relevant for understanding seasonal dynamics in wild boar distribution. We clarified this choice in the manuscript and provide references to support the use of this definition (line 211-213).

Lines 195-197: What variables were removed due to collinearity and multicollinearity? The authors state in line 213 that “Only non-collinear variables were incorporated” but these should be listed here in the methods if they were not included. Then delete Line 213? I see now these are in Table 1 (better to reference in Methods) but this table can be deleted. Simply include a sentence of which are not included because they don’t meet assumptions of collinearity/multicollinearity (i.e., opposite of Lines 328-332) in the methods not results.
A: We thank the reviewer for the helpful feedback. We have moved the description of the included variables, as well as the corresponding Table 1, into the methods section to improve clarity (lines 224-230). We acknowledge that Table 1 could seem a repetition with the text. However, we decided to maintain Table 1 as we think it provides a clear and immediate overview of the variables, making it easier for readers to reference at a glance. Keeping the table also allows us to maintain a logical and seamless flow in the result subsection "presence data and environmental variables" referring to the methods, ensuring the text remains easy to follow. We appreciate reviewer’s input in helping to enhance the clarity and linearity of the manuscript.

Lines 227-232: In your introduction, the authors discuss that SDMs can be implemented based on data available and aim of the study. However, here the authors appear to go on a habitat suitability modeling exploration with everything in Biomod2 (11 methods) which is inappropriate and unnecessary post hoc analysis that goes against the authors own introduction. Why not select the best model that fits your data and use it instead of evaluating which is best based on some criteria that we really are not clear on? More on this later.
A: We apologize for the confusion and thank the reviewer for the valuable comment. About evaluating all possible models in the Biomod2 package, we completely understand that this may seem excessive. However, since behind the choice of a model there are many considerations and assumptions, choosing the best performing model by comparing multiple ones is an accepted approach when SDMs are considered ( <https://doi.org/10.1002/ece3.6859>; <https://doi.org/10.1111/1749-4877.12000>; <https://doi.org/10.1007/s10980-021-01371-y>; <https://doi.org/10.1016/j.ecoleng.2022.106725> ). This rationale was suggested also by ENETWILD report (<https://doi.org/10.2903/sp.efsa.2018.EN-1490> ), at page 12: “*In order to provide a best possible output we would argue that all potential models should be fitted and compared to limit the risk of artefactual outputs as a result of model-specific assumptions*”. Comparing a variety of models allowed us to identify the best-performing one for our dataset. We wanted to avoid making assumptions about which models would be most suitable, and instead, allow the data to guide the selection process. We added a specification in the discussion section in line 560-561 to support our rationale.
However, we agree on the confusing introduction. Thus, we modified it accordingly, also with some references, in line 140-145.

Results

Line 341: Here the authors refer to “annual” not “overall” leading to confusion on just how these were calculated?
A: We apologize for not ensuring a uniform definition of the overall scenario throughout the manuscript. We modified the line using “overall” and thank the reviewer for helping in increasing the clarity of our work.

Line 347: I am a bit confused here. In the Introduction, the authors were recommending an “ensemble modelling approach” but here they are saying just use one, GBM? So this entire effort of evaluating all of these models was not necessary?
A: We apologize for creating confusion between introduction and the final choice of one model. We agree with your observation. The introduction can be revised to better reflect the actual approach used in the study. While we initially referred to an ensemble modeling approach, we recognize that the final model selection process was not a “real ensemble” of more algorithms, but only an ensemble of the outputs of the same model but applied in different runs. The effort of evaluating all models was necessary to ensure the robustness of the final model choice and to validate that GBM provided the most accurate predictions for this study. We updated the introduction to more accurately describe this process (line 140-145). We also revised the discussion, including as future perspective, the adoption of a “real” ensemble modelling (lines 576-579). To further minimize confusion, in line 280 we substitute “models” with “runs” when the final ensemble computation (which refers to the [BIOMOD_EnsembleModeling()](https://biomodhub.github.io/biomod2/reference/BIOMOD_EnsembleModeling.html) function according to Biomod2 package) is performed.

Line 348: See my comments above for Lines 227-232 and Line 347. The authors claim GBM was preferred due to “easier parameterization” and “more realistic performance metrics?” I am not sure how you define this or justify it being a criteria for selecting the model chosen?

A: We recognize that our phrasing may have been unclear. While we initially tested several algorithms, we ultimately selected GBM due to its balance between performance and spatial generalization across the study area. Compared to other models, GBM provided continuous suitability estimates across the study area, in association with strong predictive power in terms of evaluation metrics. RF and XGBOOST showed perfect performance metrics, approaching or equal to 1 (S2 Table), which, while mathematically optimal, warrant careful interpretation in ecological contexts, since it may be due to overfitting. We acknowledge the terms used were confusing and disorienting, thus we changed the sentence to improve the clarity adding some references in line 380-385. We also further discussed the overfitting of RF and XGBoost in the discussion section in line 562-570 . We thank the reviewer for the opportunity to clarify our choice of model.

Line 347: What is a “privileged area?”
A: By "privileged area," we intended to refer to areas that are highly suitable for wild boar presence based on our model predictions. To improve clarity, we will replace "privileged area" with "highly suitable area" in the manuscript.

Figure 1-4: These figures appear blurry and may not be up to the quality required by the journal.

A: We are sorry that the figures might seem of poor quality. We initially had high-resolution PDFs of the figures, but they exceeded the file size limits for the submission platform. To address this, we used the tool provided by the journal to reduce the file size while maintaining the best possible resolution. If needed, we are happy to provide the original high-resolution versions directly to the editorial team to ensure they meet the journal's quality standards. However, the original raster files will be available in the final version.

Figure 3: It seems to me that the suitability of habitat lines up with where the presence data is located for this region, regardless of season?

A: We thank the reviewer for the observation. The performance metrics for GBM model didn’t suggest an overfitting, while still providing good values suggesting a reliable generalization across the study area. Shifts in suitability according to seasonality were slightly observed, which might align with factors related to wild boar ecology. We added a paragraph, according also to reviewer 2 suggestion (line 498-505): “*Observed seasonal variations, such as increased suitability for wild boar presence in spring, summer, and autumn, may be linked to difference in precipitations and occurrences’ density due to reproductive cycles, food availability, and seasonal hunting regulations. For instance, a greater wild boar density in spring may be associated to higher precipitations which are linked to more food resources. Higher wild boar activity in spring and summer may correspond to the birth of piglets, increased movement in search of food, and reduced hunting pressure, which typically occurs in autumn and winter. However, also human-related reporting biases must be considered*.”.

Additionally, areas without presence data showed high suitability, indicating that the model isn’t simply mirroring presence locations.

However, we acknowledge that our seasonal maps represent a preliminary attempt to assess seasonal variability in landscape suitability. The variable "precipitation," the only season-sensitive factor in the final models, emerged as a key variable only for the winter season, as described in line 548-550. Consequently, any interpretation of seasonal differences remains speculative. As such, we decided to present the seasonal maps as supplementary results (S1 Fig), encouraging further improvements for the future. This is discussed in more detail in lines 548–559. Now, Fig 3 shows only the overall scenario, while previous Figure 3 with also seasonal maps, is included as S1 Fig.

Figure 4: It is difficult to determine this figure for panel A. I suggest you zoom in on the connectivity map like in B and C but replace those panels with connectivity values instead of binary risk?
A: We modified the figure according to reviewer suggestion.

Discussion

Lines 4342-436: I am not sure the authors succeeded here based on my point above that the suitability and connectivity seem to follow the presence data? Using a 100 m resolution also seems too fine a scale considering the size of their study area. There may be too similar characteristics across the study area at the 100 m scale thus masking the variables that were really influencing wild boar distribution across your landscape? A larger scale, with your variables summarized to each raster cell (500m or 1000 m) might provide more value and identify more influential variables in your models?

A: We thank the reviewer for the suggestion. However, our decision to use this finer scale was deliberate, as we aimed to provide detailed, high-resolution maps that could offer more localized insights for management and conservation efforts, also considering the specific characteristics of the study area. In particular, the use of a 100 m resolution can enhance the likelihood of capturing fine-scale environmental variability relevant to wild boar habitat preferences and connectivity in Northern Italy. Below, we justify the use of a high-resolution scale with some literature.

In areas with complex topography and ecological diversity, like Northern Italy, fine-scale environmental heterogeneity significantly affects species habitat preferences and movement patterns. High-resolution data (e.g., 100 m) offer several advantages, related to the detection of small-scale habitat features, barriers, and corridors critical for wild boar (Cahill et al., 2003; Massei & Genov, 2004). For instance, high-resolution data better capture Northern Italy’s landscape variability (mountains, valleys, mixed land cover), accurately reflecting the landscape features that influence species presence, such as elevation, slope, vegetation, and human infrastructure. Regarding landscape connectivity, in fine-scale heterogeneous landscapes, wild boar movement is influenced by local conditions. Connectivity models using 100 m resolution data can identify small-scale corridors and barriers crucial for wildlife movement (Zeller et al., 2012). Therefore, coarser resolutions, such as 500 m or 1000 m, may obscure these details by smoothing landscape variation, potentially overlooking important microhabitats and landscape resistance factors (Guisan & Thuiller, 2005; Zeller et al., 2012).

We acknowledge the concern about potential redundancy in environmental variables at a 100 m scale. However, we pre-processed the variables using statistical techniques such as variance inflation factor (VIF) analysis to ensure minimal redundancy and maximize the ecological relevance of the selected variables. This methodology allows us to leverage high-resolution data while minimizing multicollinearity, providing a detailed and reliable prediction of suitability and connectivity across a complex landscape.

Nevertheless, despite our current 100 m resolution aligns well with the environmental and ecological dynamics we aimed to capture, future studies could explore multi-resolution approaches. We added this future perspective in lines 532-535.

-----------

Cahill, S., Llimona, F., & Gracia, J. (2003). Spacing and nocturnal activity of wild boar Sus scrofa in a Mediterranean coastal area. Wildlife Biology, 9(1), 29-36.

Guisan, A., & Thuiller, W. (2005). Predicting species distribution: offering more than simple habitat models. Ecology Letters, 8(9), 993-1009.

Massei, G., & Genov, P. V. (2004). The environmental impact of wild boar. Galemys, 16(1), 135-145.

Zeller, K. A., McGarigal, K., & Whiteley, A. R. (2012). Estimating landscape resistance to movement: a review. Landscape Ecology, 27(6), 777-797.

Lines 442-449: Agreed, does the lack of low suitability in Po Valley make sense for how wild boar select habitat in Europe? Are there any detailed VHF or GPS data collected in this region to determine if wild boar prefer agriculture-forest valleys compared to forested, mountainous terrain?
A: The results presented in our study are consistent with previous estimations, which showed lowest suitability value in the Po Valley, although at lower resolutions (references: <http://dx.doi.org/10.5334/ohd.24>; <https://doi.org/10.1111/tbed.12559>). However, currently, there are no VHF or GPS radiotracking data available for wild boar in this region to assess their precise preferences (at least publicly) between the different landscapes. Collecting such data in the future would indeed be valuable, as it could provide a more precise understanding of habitat selection and movement patterns across diverse landscapes. We added this point in the discussion in line 556-559 and thank the reviewer for having highlighted this future perspective.

Lines 484-486: The authors acknowledge the temporal pattern but it was not clear how these were modeled with “time” as stated in the methods? All climate variables are available daily, weekly, monthly correct? However, the authors used a static annual mean or 3-4 months seasonal mean which likely rendered these variables of limited value in their assessment. These climate variables and NDVI could have avoided these pitfalls the author outline if they were evaluated differently here?

A: While it is true that climate variables are available at more granular temporal resolutions (daily, weekly, and monthly), our decision to employ annual or seasonal means was driven by the objective of focusing on broader climate trends relevant to wild boar habitat suitability. We added a sentence in the discussion in line 556-559 to recognize that a more dynamic approach, utilizing daily or weekly variables, could provide a richer understanding of how seasonal variability may influence habitat selection over time. We thank the reviewer for highlighting this important aspect, as it encourages us to refine our methodologies and consider more nuanced evaluations in subsequent studies.

**Reviewer #2:** Faustini et al. presented a ms entitled “Habitat suitability mapping and landscape connectivity analysis to predict African swine fever spread in wild boar population: a focus on Northern Italy” for publication in PLOS ONE. The paper deals with a relevant and interesting topic: a highly contagious disease affecting wild and domestic pigs (ASF) reported in Eastern Europe and, in January 2022, in North-western Italy. Thereafter, the expanding wild epidemic, has posed serious management problems related to the presence of Italian pig farms and trade.

I enjoyed reading this ms and I think that this study is a valuable contribution to improve the knowledge/management of this species.
However, despite the interesting topic and the potential of management implications for the target species (i.e., mapping of suitable habitats for wild boars and their potential dispersal corridors in Northern Italy, could be an important issue in preventing the ASF expansion), I believe that a revision is required (please, see my comments below).

A: We deeply appreciate the reviewer’s recognition of the relevance of our work and thank the reviewer for the positive general feedback on our manuscript. We are committed to further improving the manuscript based on reviewer comments and suggestions.

General comments:

1) A first flaw that I found concerns the absence of an accurate description of ASF Genotype II expansion (speed, population crash, corridors) in the Eastern European countries. I believe this is a fundamental point: to better introduce the issue, the authors should describe the situation of the last 10 years in Eastern Europe in detail, also in relation to the economic impact of this epidemic.

A: In response to the reviewer’s comment, we have modified the manuscript to include more detailed information on the expansion of ASF Genotype II in Eastern Europe. A new paragraph has been added that covers the speed of the disease's spread, its impact on wild boar populations, and the economic effects on the pork market. Additionally, we tried to emphasize more the distinction between the ASF spread through wild boar movements and human activities, making these mechanisms clearer in the text. To improve the readability and flow, we have also made slight adjustments to the following paragraph. We sincerely appreciated the reviewer’s suggestions, as they have significantly helped improve the comprehensiveness of the manuscript.

2) I think the ms would gain more value with a reorganization of the discussion section. I suggest dealing first with the description of the choices made by wild boar, then the description of the potential expansion corridors (with details on the most important directions and their characteristics) and finally an analysis of the possible management and surveillance interventions. These three parts are already present in the discussion but should be better outlined, especially the part related to connectivity.

A: We thank the reviewer for the helpful feedback. We acknowledge that the length and structure of the discussion could impact its clarity and flow. To enhance readability, we have added subtitles to the discussion to clearly delineate the sections on wild boar habitat selection (“Northern Italy suitability for wild boar”), potential expansion corridors (“Northern Italy main corridors of wild boar dispersal”), and the “implications for ASF management and surveillance strategies”. Additionally, we have merged the last lines related to the seasonal ASF cases pattern into the initial part (line 506-512). We hope these changes will make the discussion more accessible and easier to navigate.

3) A detailed explanation/comparison of seasonal models is also missing. How does expansion relate to seasons?

A: A more detailed discussion on results obtained with seasonal model was added in lines 498-505. Additionally, we have merged the last lines related to the seasonal ASF cases pattern into the initial part (line 506-512). We thank the reviewer for the comment as it allowed us to add value to our discussion section.

4) The manuscript often reports the names of valleys, rivers, mountain areas with reference to the figures (e.g., Fig. 3 - L. 442-446) but these are not reflected in the maps. For a reader who is not familiar with these areas, it is difficult to interpret the results. Supplementary material with more detailed maps could be added.

A: We apologize for the lack of clarity. We added a Supplementary Figure 2 (S2 Figure) reporting the areas referred in the lines mentioned by the reviewer. We thank the reviewer for the suggestion, as it helped to improve the interpretation and clarity of results.

5) Also the “discontinuous natural zones” (L. 471) should be represented in a map, according to their importance in connectivity
A: We modified “discontinuous natural zones” with “discontinuous natural parks”, adding the reference to the S2 Figure, to improve the clarity.

Minor comments:

Abstract: I suggest to delete the description of ASF Genotype I that is not the target of this ms.

A: We thank the reviewer for the suggestion, and we modified the abstract accordingly.

L.439-441: “In addition to the higher resolution of the maps presented here compared to those found in the literature, the influence of each variable on wild boar presence was also detailed.”
Please include the references of the studies compared with the results obtained in this research.
A: We apologize for the oversight. The proper references were included (line 482).

L. 507-510: I think that high-traffic corridors need to be described: characteristics, directions and relationships with existing farms.

A: With the term of “high-traffic corridors” we mean multiple linked areas (pixels) with high connectivity value in the obtained connectivity map. We added this specification along with more details on corridors directions in lines 589-601. Estabilishing the relationship with existing farms was out of the aims of this study. However, we included it as future perspectives in line 601-603.

6. PLOS authors have the option to publish the peer review history of their article ([what does this mean?](https://c05y1x9s.r.us-east-2.awstrack.me/L0/https:%2F%2Fjournals.plos.org%2Fplosone%2Fs%2Feditorial-and-peer-review-process%23loc-peer-review-history/1/010f0191fe7c8ab3-52ad4259-c444-4a12-92fc-9b77ef218fa2-000000/r-OB9laH3Bwl6H8Mne3-pvC2wL4=176)). If published, this will include your full peer review and any attached files.


If you choose “no”, your identity will remain anonymous but your review may still be made public.


**Do you want your identity to be public for this peer review?** For information about this choice, including consent withdrawal, please see our [Privacy Policy](https://c05y1x9s.r.us-east-2.awstrack.me/L0/https:%2F%2Fwww.plos.org%2Fprivacy-policy/1/010f0191fe7c8ab3-52ad4259-c444-4a12-92fc-9b77ef218fa2-000000/wH2fdsYZNVKpneZIGnZBZAeHCK8=176).

Reviewer #1: No

Reviewer #2: No
