# Supplementary material for: Habitat suitability mapping and landscape connectivity analysis to predict African swine fever spread in wild boar populations: A focus on Northern Italy
Source: PLoS One. 2025 Jan 30;20(1):e0317577. doi: 10.1371/journal.pone.0317577 (PMC11781678; doi:10.1371/journal.pone.0317577)
Supplement: Attachment — Submitted filename: Response to Reviewers.docx [file pone.0317577.s010.docx]

**Response to Reviewers**

Journal Requirements:

Please review your reference list to ensure that it is complete and correct. If you have cited papers that have been retracted, please include the rationale for doing so in the manuscript text, or remove these references and replace them with relevant current references. Any changes to the reference list should be mentioned in the rebuttal letter that accompanies your revised manuscript. If you need to cite a retracted article, indicate the article’s retracted status in the References list and also include a citation and full reference for the retraction notice.

A: We thank for the reminder to review the reference list. We confirm that the manuscript does not contain any retracted papers. Based on Reviewer 1's comments, we have updated reference 67 (now reference 60) to accurately reflect the correct source. The details of this modification are described in our response to Reviewer 1.

**Additional Editor Comments:**

Dear Authors,

I would like to thank you for considering and addressing most of the reviewers' comments. I believe the manuscript contributes significantly to the understanding of a very interesting and sensitive subject.

I must say that given the wide range of potential applications for suitability models, there may never be a "perfect" model or an entirely "wrong" one. Furthermore, due to the complexities of wild boar ecology, modeling its distribution will always present challenges. This species in Europe can be found in very different habitats, ranging from urban areas to mountainous regions up to 2000 m a.s.l. Initially, as Reviewer 1, I was skeptical about the use of NDVI data averaged over a ten-year period. However, I now realize that investigating the subject in greater detail would involve a vast array of additional covariates (e.g., beech and chestnut masting, snow cover, predator densities, hunting pressure), making the endeavor endless. Thus, I consider your selection of covariates appropriate.

A: We thank the Editor for the kind words and the encouraging feedback. We appreciate the recognition of the challenges inherent in modeling the distribution of such an adaptable and widely dispersed species as the wild boar.

However I would like to request a few additional improvements:

As suggested by Reviewer 1, please carefully confirm the number of variables used in the modeling process 11 or 13?

A: Thank you for pointing this out. For each scenario, 13 variables were selected for the modeling process, 11 of which were common across all scenarios. We apologize for the misunderstanding and have revised the manuscript accordingly to make this point clearer (lines 225-230). We appreciate editor and reviewers’ attention and hope this resolves the confusion.

Additionally, I agree with Reviewer 1 that Table 1 does not add substantial value to the main text and should be moved to the supplementary material.

A: We now acknowledge that Table 1 does not add substantial value to the main text. Accordingly, we have moved Table 1 to the supplementary material as recommended (now S1 Table). We appreciate Editor and reviewer 1 input, which has helped improve the clarity and focus of the manuscript.

Please carefully review the references, as suggested by Reviewer 1. For example, while working with grids in R, I currently use the "Terra" package, but I understand that the "Raster" package is still functional for older versions of R. Kindly verify which package was utilized and ensure the references reflect this accurately.

A: We thank the editor for highlighting the importance of accurate referencing. Upon review, we realized that there was an oversight in our citation. We sincerely apologize for the confusion.

The functions we utilized for the correlation tree belong to the *stats* package in R, and not the *Raster* package as previously cited. We have corrected this in the manuscript and updated the references accordingly. Additionally, we have verified all other references to ensure their accuracy.

We greatly appreciate editor and reviewer 1 attention to detail, which has helped us improve the quality and precision of our work.

While reading the manuscript, I felt that the seasonal models were not well integrated into the overall narrative. It would be helpful to clarify why you chose to model seasonal distributions and to better explain why the finding of increased suitability in spring could be of any interest. Does this have any management implications?

A: We thank the editor for the observation and have revised the manuscript to better explain the rationale for modeling seasonal distributions.

We chose to model seasonal distributions to account for the variations in habitat use and resource availability for wild boars across seasons. We specified this choice in materials and methods section, in the lines 205-207 and lines 233-234. Specifically, the finding of increased suitability in spring suggests a period of heightened activity and resource exploitation, which is likely influenced by factors such as vegetation growth and reproductive cycles (line 494-500).

From a management perspective, these findings could inform targeted strategies, such as timing interventions to optimize hunting schedules, disease surveillance plans, and the implementation of biosecurity measures. To clarify this connection, we have added this specification to line 500-502 in the revised manuscript (discussion section).

We appreciate Editor feedback, which has allowed us to strengthen the narrative and highlight the practical implications of our findings.

[Note: HTML markup is below. Please do not edit.]

Reviewers' comments:

Reviewer's Responses to Questions

**Comments to the Author**

1. If the authors have adequately addressed your comments raised in a previous round of review and you feel that this manuscript is now acceptable for publication, you may indicate that here to bypass the “Comments to the Author” section, enter your conflict of interest statement in the “Confidential to Editor” section, and submit your "Accept" recommendation.

Reviewer #1: (No Response)

Reviewer #2: All comments have been addressed

2. Is the manuscript technically sound, and do the data support the conclusions?

The manuscript must describe a technically sound piece of scientific research with data that supports the conclusions. Experiments must have been conducted rigorously, with appropriate controls, replication, and sample sizes. The conclusions must be drawn appropriately based on the data presented.

Reviewer #1: Partly

Reviewer #2: Yes

3. Has the statistical analysis been performed appropriately and rigorously?

Reviewer #1: No

Reviewer #2: Yes

4. Have the authors made all data underlying the findings in their manuscript fully available?

The [PLOS Data policy](http://track.editorialmanager.com/CL0/http:%2F%2Fwww.plosone.org%2Fstatic%2Fpolicies.action%23sharing/1/010f019394405959-96f54071-69cd-47bc-a8ab-52ba7505d433-000000/dyTumVuyVjNwUn-tNqVGBCQOBz4tbCBUHtXqOmuRaIM=187) requires authors to make all data underlying the findings described in their manuscript fully available without restriction, with rare exception (please refer to the Data Availability Statement in the manuscript PDF file). The data should be provided as part of the manuscript or its supporting information, or deposited to a public repository. For example, in addition to summary statistics, the data points behind means, medians and variance measures should be available. If there are restrictions on publicly sharing data—e.g. participant privacy or use of data from a third party—those must be specified.

Reviewer #1: No

Reviewer #2: Yes

5. Is the manuscript presented in an intelligible fashion and written in standard English?

PLOS ONE does not copyedit accepted manuscripts, so the language in submitted articles must be clear, correct, and unambiguous. Any typographical or grammatical errors should be corrected at revision, so please note any specific errors here.

Reviewer #1: Yes

Reviewer #2: Yes

6. Review Comments to the Author

Please use the space provided to explain your answers to the questions above. You may also include additional comments for the author, including concerns about dual publication, research ethics, or publication ethics. (Please upload your review as an attachment if it exceeds 20,000 characters)

Reviewer #1: I appreciate the effort of the authors to pull together this information, the manuscript has been improved considerably in the Introduction specifically, and I appreciate the authors efforts in addressing reviewer concerns.

A: We thank the reviewer for the kind words, and we are pleased to read that the revisions have significantly improved the manuscript. We appreciated reviewer 1's thoughtful comments and constructive suggestions, which were instrumental in enhancing the quality of the manuscript.

There were still a few concerns that were not addressed and one that I overlooked that I will go into detail below:
A: We thank the reviewer for the comments. We carefully considered the points that reviewer 1 has raised, and provided a detailed response to each one below, trying to meet each request.

Materials and methods

Lines 215-217: What is reference 67 actually referring to here? There is no mention of a correlation tree or cluster dendrogram or raster package in the document? How can the raster package have been used when it was removed from CRAN for use in program R in December 2023? It appears the authors are citing a review (Anderson; Journal of Experimental Marine Biology and Ecology 289(2):303–305) of the book:

Analysis of Ecological Communities: Bruce McCune and James B. Grace, MjM Software Design, Gleneden Beach, USA, 2002, ISBN 0 9721290 0 6, US$ 35 (Pbk) May 2003.

Regardless, none of these terms appear in the book either as far as I can tell.

A: We thank the reviewer for pointing out the oversight regarding the reference now on lines 216–218. The reviewer is absolutely correct that there was an error in our citation. We sincerely apologize for the mistake and any confusion it may have caused.

The functions used for the correlation tree actually belong to the *stats* package in R, and we have corrected the manuscript to reflect this (line 218). As reported by “> citation("stats")” function: “The ‘stats’ package is part of R. To cite R in publications use: R Core Team (2023). _R: A Language and Environment for Statistical Computing_. R Foundation for Statistical Computing, Vienna, Austria. <https://www.R-project.org/>.”

Additionally, we have included the appropriate reference for the Variance Inflation Factor (VIF) to enhance reproducibility (line 221).

We are grateful for the reviewer’s careful review and for bringing this to our attention, allowing us to improve the accuracy and clarity of our work.

Line 224: “The variables included in the study for all scenarios were 11:…” Do the authors mean “overall” here instead of “all” to align with text and table 1 that refers to all seasons combined (Line 207)?
A: We apologize for the potential confusion regarding the use of "all" versus "overall". In the previous review, we verified that the term "overall" was consistently used to avoid ambiguity. In this section, we decided instead to retain "all" because it specifically refers to the fact that for each scenario, 13 variables were selected for the modeling process, 11 of which were common across all scenarios. Table 1 (now S1 Table) served instead as a visual summary to complement this explanation.

We apologize for any misunderstanding and have revised the manuscript to avoid further misunderstandings (line 225).

Lines 224-228: In my previous review, I suggested “Simply include a sentence of which are not included because they don’t meet assumptions of collinearity/multicollinearity (i.e., opposite of Lines 328-332) in the methods not results.” The authors chose to not edit with my recommendations and also chose not to delete Table 1. Perhaps this time around I might provide a more compelling argument to deleting (or modifying) Table 1 due to the number of issues with it’s inclusion:

A: We thank the reviewer for the thoughtful feedback and for providing a more detailed explanation regarding his/her previous suggestion. We apologize for not fully addressing the reviewer’s earlier recommendation, and we appreciate his/her patience in this matter.

We now understand the importance of simply including a sentence in the methods section to explain which variables were excluded due to assumptions of collinearity/multicollinearity. We have revised the manuscript accordingly, incorporating this clarification as suggested in lines 225-230.

Regarding Table 1, we have reconsidered your suggestion, and, after further evaluation of editor’s comment, we have decided to move Table 1 to the supplementary material to better align with the focus of the main text.

We are grateful for reviewer constructive comments, which have been instrumental in improving the clarity and quality of our manuscript.

1. Out of 20 variables, 11 were acceptable to include in the overall analysis although Table 1 has x in 13 columns along with the total that confirms the 13 and not the 11?

A: We are sorry for the confusion. To clarify, the 11 variables refer to those common across all models, including both overall and seasonal scenarios. The 13 variables, as indicated in Table 1 (now S1 Table), represent the total number of variables considered for each scenario, with 11 of them being shared across all models.

We modified the manuscript according to reviewer and editor feedbacks (line 225). We hope this clears up the confusion.

2. The authors list the 11 here as well as several that were not included in overall or seasonal models which, in turn, is presenting "results" in the Materials and Methods? There is also a section in the Results (Lines 365-368) that says out of the 20 variables that 13 were selected in the overall and seasonal suitability models that references Table 1. It is not clear why some of this analysis is in the Materials and Methods and some is in Results?
A: We understand the concern regarding the placement of certain details in the Materials and Methods and in the Results sections. To clarify, both sections refer to the same analysis, not to two different analyses. We simply decided to include a brief sentence in the Results section to describe the results of the selection of variables, as we aimed to present the process of variable choice for clarity, transparency and reproducibility. We acknowledge that this might seem repetitive. However, by placing and recalling this information in the Results, we hoped to maintain a logical flow of the modeling process, making it easier for readers to follow the progression of the analysis. We specified that the collinearity/multicollinearity analysis refers to the one described in material and methods section in line 362.

We hope this explanation clarifies our approach.

3. Lines 226-228 identifies several variables that were excluded in all scenarios (overall and seasonal, I presume), and interestingly, 2 of the variables (Temperature and NDVI) are those averaged over a 10 year span that I mentioned was not appropriate in my previous review (see below comments from my previous review and author response).

A: We thank the reviewer for providing additional arguments. We modified the manuscript according to the reviewer and editor’s suggestions. We believe this revision will streamline the manuscript and better align with the flow of the content (see below).

4. Table 1 repeats some of what is in text in both sections and I would still suggest that it be deleted. If the authors believe it has value, then why not include mean (SDs) for each variable during each season and overall instead of just an x? An entire page for a table with x’s that could be included in 4 sentences in the Materials and Methods (like Lines 226-228 so one line for overall and one line for each season) does not seem necessary and I don’t see how it helps the “flow” considering it is also presented in the Results?

A: We really appreciate reviewer effort in providing such detailed arguments. We thank the reviewer for the thoughtful comments and suggestions. After considering your arguments, we now acknowledge the need to remove the table from the main text. As suggested by the editor, we moved Table 1 in the supplementary material. We agree that the information it contains can be succinctly presented in the text, as you suggested, and that the table may not add significant value to the manuscript's flow.

We appreciate reviewer input, which has helped us improve the manuscript's clarity and structure.

From my previous review:

Lines 484-486 (original submission): The authors acknowledge the temporal pattern but it was not clear how these were modeled with “time” as stated in the methods? All climate variables are available daily, weekly, monthly correct? However, the authors used a static annual mean or 3-4 months seasonal mean which likely rendered these variables of limited value in their assessment. These climate variables and NDVI could have been match with each week-month-year presence data were recorded?

To which the authors responded:

A: While it is true that climate variables are available at more granular temporal resolutions (daily, weekly, and monthly), our decision to employ annual or seasonal means was driven by the objective of focusing on broader climate trends relevant to wild boar habitat suitability. We added a sentence in the discussion in line 556-559 to recognize that a more dynamic approach, utilizing daily or weekly variables, could provide a richer understanding of how seasonal variability may influence habitat selection over time. We thank the reviewer for highlighting this important aspect, as it encourages us to refine our methodologies and consider more nuanced evaluations in subsequent studies.

Reviewer second response: Again, I suggested the authors assign temperature and NDVI to the monthly or even seasonal means instead of a 10 year mean (overall or seasonal). The authors identified, in their response and in the manuscript that a “more dynamic approach, utilizing daily or weekly variables, could provide a richer understanding…” however the authors chose not to do it in their revision? Instead, they argue how it can be done in the future? Furthermore, do the authors realize that both Temperature and NDVI were not included in any models (Based on Table 1 multicollinearity) and Precipitation was include in 3 of the 5 scenarios only? Regardless of what past research claims, the authors have the ability to match these variables to the annual/seasonal means to match them to the data but chose not to do so. The authors might be better off removing them entirely in the manuscript and models considering they provide no value based on how they were presented in the models (perhaps due to static 10 year averages)? Based on how you present them in Table 1, Temp and NDVI were not included in any models, correct?

A: We appreciated the reviewer’s detailed feedback and thank him/her for further emphasizing the importance of the temporal resolution of climate variables. We understand your concerns and would like to clarify our approach regarding temperature, NDVI, and precipitation.

For the seasonal scenarios, precipitation, temperature and NDVI were processed to obtain seasonal means (i.e., a 3-month average), as explained in lines 207-209 of the manuscript. This was done to align the temporal scale of the climate variables with the seasonal dynamics of wild boar habitat suitability.

It is correct that temperature and NDVI were not ultimately included in the models due to collinearity with other variables that we deemed more closely related to wild boar ecology and easier to interpret. We acknowledged that these other variables (finally included) were less processed, and more directly linked to habitat suitability. However, we did not remove temperature and NDVI entirely from the study, as we believe their inclusion in the analysis serves as a useful reference for future research.
Furthermore, our study suggests that seasonal NDVI and Temperature variability might be captured by other variables. The lack of significance of temperature and NDVI in the final models is, in itself, a meaningful ecological result. The exclusion of these variables highlights that their influence on habitat suitability for wild boars may be effectively represented or overshadowed by other, more directly relevant variables. Including or excluding temperature and NDVI in the final models would not have altered the model’s predictive capacity. We specified this concept in lines 517-520.

By presenting our full study design, we aimed also to provide guidelines for more detailed and dynamic analyses in subsequent studies.

Moreover, presenting also not included variables encourages future studies to consider the incorporation of more detailed variables linked to Temperature and NDVI (i.g., availability of seasonal food resources) for a more comprehensive understanding of habitat suitability (line 555-556).

We appreciate reviewer valuable insights, which have contributed to refining the manuscript and better articulating our approach, as well as the limitations and directions for future research.

Supporting information, S1 Files: This Gitlab repository does not exist or is not accessible to the reviewer. The authors agreed that it should be available “upon acceptance,” however, not available to the reviewer? It seems that accepted or not, a GitLab repo would be valuable to document your work and provide this analysis as an archive for your efforts. Including this link in your revisions has little value if a reviewer is not able to access it?

A: We apologize for the inconvenience caused by the repository not being accessible during the review phase. We understand that including an unfunctional link in the revisions adds no practical value. We already included the link to the repository at this stage based on the previous editor’s suggestion to help accelerate the process after acceptance.

As per PLOS ONE’s policy, the repository will be made publicly accessible after the manuscript’s acceptance. We appreciate your understanding and would be sincerely happy to provide access as soon as the paper is accepted for publication by the editor.

Reviewer #2: This revised version has improved a lot and accounted for most of the problems I identified when reading the first version.
I think the authors have made a considerable effort to increase the quality of their ms.

A: We sincerely thank Reviewer 2 for the positive feedback and for acknowledging the efforts made to address the issues raised in the initial version. We deeply appreciate reviewers’ time and expertise in reviewing our work.

7. PLOS authors have the option to publish the peer review history of their article ([what does this mean?](https://track.editorialmanager.com/CL0/https:%2F%2Fjournals.plos.org%2Fplosone%2Fs%2Feditorial-and-peer-review-process%23loc-peer-review-history/1/010f019394405959-96f54071-69cd-47bc-a8ab-52ba7505d433-000000/3WfqafnnY6TKInsZfi0yEFyxa-zQ4VzAvXrylfICvG0=187)). If published, this will include your full peer review and any attached files.


If you choose “no”, your identity will remain anonymous but your review may still be made public.


**Do you want your identity to be public for this peer review?** For information about this choice, including consent withdrawal, please see our [Privacy Policy](https://track.editorialmanager.com/CL0/https:%2F%2Fwww.plos.org%2Fprivacy-policy/1/010f019394405959-96f54071-69cd-47bc-a8ab-52ba7505d433-000000/j9Cjz9gBFpMBqxTDf6Putehiyze1Vqc9RFmfM8qebdM=187).

Reviewer #1: No

Reviewer #2: No
